# Pilot study on the population genetics structure of *Fasciola hepatica* from seven provinces of South Africa

**DOI:** 10.3389/fvets.2025.1659523

**Published:** 2025-09-11

**Authors:** Sophy Nukeri, Mokgadi P. Malatji, Clearance M. Mnisi, Mamohale Chaisi, Samson Mukaratirwa

**Affiliations:** ^1^School of Life Science, College of Agriculture, Engineering and Science, University of KwaZulu-Natal, Durban, South Africa; ^2^Research and Scientific Services Directorate, National Zoological Garden, South African National Biodiversity Institute, Pretoria, South Africa; ^3^Department of Environmental, Water and Earth Sciences, Faculty of Science, Tshwane University of Technology, Pretoria, South Africa; ^4^Department of Veterinary Tropical Diseases, University of Pretoria, Pretoria, South Africa; ^5^Center for Zoonoses and Tropical Veterinary Medicine, Ross University School of Veterinary Medicine, Basseterre, Saint Kitts and Nevis

**Keywords:** genetic diversity, population structuring, cattle, *Fasciola hepatica*, microsatellites, South Africa

## Abstract

**Introduction:**

Fasciolosis is a neglected tropical disease caused by *Fasciola hepatica* and *Fasciola gigantica,* affecting livestock, wildlife, and humans globally. Understanding the genetic diversity and population structure of *Fasciola* spp. is essential for tracking transmission patterns, detecting drug resistance, and guiding targeted control efforts. In South Africa, where such data are lacking, this study provides critical insights to support evidence-based interventions. This study aimed to assess genetic structure of *Fasciola* populations collected from cattle across seven provinces in South Africa.

**Methods:**

Liver flukes were collected from 57 cattle from 13 provincial abattoirs across South Africa, and DNA was extracted from 189 *F. hepatica* specimens. Although sampling efforts varied slightly due to abattoir throughput, efforts were made to maximize geographic and ecological representation. Six polymorphic microsatellite loci were selected to assess genetic diversity based on their broad allelic range and prior validation for *F. hepatica* population genetic studies. The allele frequencies, F*is* and F*st* values, heterozygosity, and genetic distances were calculated on GenAlEx 6.51b2. Structure 2.3.4 was used to detect population structure.

**Results:**

A total of 277 alleles were identified across loci, with allelic richness varying by province. All loci were polymorphic, and the mean number of alleles varied from 3.667 to 9.667. Moreover, out of the total number of alleles identified, 3% were private alleles. Observed heterozygosity (Ho) ranged from 0.182 to 1.000, while the expected heterozygosity (He) ranged from 0.165 to 0.899. These ranges suggest differences in genetic diversity and potential inbreeding or population structure across the samples studied. The fixation index (F) value ranged from −0.017 to 0.426. F*st* values varied from 0.064 to 0.107, suggesting moderate genetic differentiation between the populations, and the number of migrants per generation (Nm) varied between 2.080 and 3.898, with an average of 3.173, which indicated a high gene flow between provinces.

**Conclusion:**

The Ho and He indicated moderate genetic diversity within populations, while the *F*-value showed moderate differentiation among populations. Geographic structuring of populations was observed, with the STRUCTURE and principal coordinate analysis (PCoA) revealing four distinct genetic clusters across seven provinces. Mpumalanga and Gauteng provinces displayed high genetic diversity and a high number of private alleles, suggesting potential reservoirs of genetic variation. Genetic distances varied by region, with neighboring provinces showing lower genetic distances, indicating gene flow and genetic connectivity across regions, which might be supported by the movement of livestock for trade. These findings highlight the genetic complexity and potential epidemiological challenges for fasciolosis in South Africa. Furthermore, considerable genetic diversity and gene flow across regions may complicate fasciolosis control and surveillance efforts in South Africa.

## 1 Introduction

Fasciolosis is a neglected tropical disease affecting humans, domestic, and wild animals, caused by food- and water-borne trematodes, *Fasciola hepatica* (Linnaeus, 1758) and *Fasciola gigantica* (Cobbold, 1856) (1). Various climatic regions, ecological conditions, and availability of snail intermediate hosts (IHs) determine the geographical distribution of both trematodes. *Fasciola hepatica* has the widest distribution, occurring on five continents (2), where it utilizes *Galba truncatula* (Müller, 1774) as the main intermediate host (IH) (3). *Fasciola gigantica* occurs in the tropical regions of Asia and Africa (4), where it has adapted to the *Radix* species of the “*auricularia* super-species” (Hubendick, 1951), *Radix rubiginosa* (Minchelin, 1831) in Asia, and *Radix natalensis* (Krauss, 1848) in Africa (5, 6) as IHs. Regions where conditions support the establishment of IHs of both flukes have reported the presence of hybrid or intermediate forms of *Fasciola,* particularly in Africa (7–14) and Asia (15–19).

Various processes, including gene flow and genetic drift, that interact with each other and influence the selection of adaptive traits shape genetic diversity (20). Thus, the extent of such processes can be studied through patterns of population genetic structure, which can be used to develop hypotheses on the evolution of traits such as drug resistance (21–23). Gene flow and genetic drift are the two main processes that generally have opposing effects on population genetic structure and further determine the efficacy of selecting favorable mutations over many generations (20). For example, in large populations with high gene flow, the effective population size (Ne) increases thus leading to smaller random fluctuations in allele frequency. Moreover, increased gene flow between populations enhances the spread of mutations. However, gene flow may slow down local adaptations when selection pressures vary among populations (20). Understanding the balance between these processes is crucial to interpret observed genetic patterns and predicting the evolutionary pathway of traits under selection.

Despite the importance of population genetic structure studies for efficient management of parasitic diseases and in offering insights into evolutionary processes and transmission dynamics (24), information regarding levels of genetic variability and genetic differentiation between *F. hepatica* populations is scanty in South Africa. The scarcity of knowledge hinders the development of targeted control strategies, which is notable considering that incidences of bovine fascioliasis have been documented in several provinces of the country including Mpumalanga and KwaZulu-Natal provinces, where both *F. hepatica* and *F. gigantica* occur and novel haplotypes have been described (25, 26). Infections have also been reported in wildlife and other non-ruminant mammals (27), underscoring the broad host range of the parasite. Although human cases are rare in the country, clinical reports dating back to the 1950s and 1960s confirmed *F. hepatica* infection associated with hepatobiliary disease (28, 29) and a more recent case described by Black et al. (30). Moreover, studies from several European countries reported the emergence of anthelmintic resistance by the liver flukes (31–33). South Africa has a unique scenario, with the presence of both *F. hepatica* and *F. gigantica*, as well as the invasive snail IH, *Pseudosuccinea columella* (34), which transmits both species (35, 36) and may facilitate hybridization (25, 26). Most molecular studies of *Fasciola* spp. in South Africa have been based on the genetic markers of mitochondrial (mt) Cytochrome c oxidase subunit I (COI) and/or ribosomal ITS (25, 26). However, the use of these mtDNA markers has limitations in population genetic structure studies (20, 37) as mtDNA generally lacks recombination. This makes the inference of dispersal patterns difficult from the analysis of variation among intrapopulations if the identification of clones is required (20). In contrast, microsatellites offer higher polymorphism and are more appropriate for detecting fine-scale genetic differentiation. Hence, this study employed microsatellite DNA markers to determine the population genetic structure of *F. hepatica* from seven provinces, covering eight different agro-climatic regions of South Africa. This is important for efficient management of parasitic diseases and offers insights into evolutionary processes and transmission dynamics (24).

The specific objectives were to evaluate genetic diversity within and among *F. hepatica* populations based on allelic richness, heterozygosity, and fixation indices and furthermore assess genetic differentiation and population structure of *F. hepatica* using F-statistics and Bayesian clustering approaches.

## 2 Methods

### 2.1 Ethical consideration

The protocols for this study were approved by the University of KwaZulu-Natal Ethics Committee (AREC/020/020PD), the South African National Biodiversity Institute National Zoological Gardens Animal Research Ethics and Scientific Committee (SANBI/RES/P2021/10), and the Department of Agriculture, Land Reform and Rural Development Section 20 permit (12/11/1/1/18 (1866)). Consent letters, for post-mortem examination of livers where flukes or signs of liver damage are visible, were obtained from abattoir managers.

### 2.2 Study sites and sample collection

One hundred and eighty-nine (*n* = 189) adult *Fasciola* parasites were opportunistically collected from livers of 57 infected cattle from seven provinces in South Africa, namely, Gauteng (84), Mpumalanga (35), Free State (13), North West (11), Northern Cape (13), Eastern Cape (17), and KwaZulu-Natal (16). The cattle were slaughtered at 13 abattoirs from seven provinces in South Africa (Gauteng = 21, Mpumalanga = 13, Free State = 4, North West = 4, Northern Cape = 3, Eastern Cape = 4, KwaZulu-Natal = 8). Specimens were preserved in 70% ethanol prior to transportation to the laboratory at the Pretoria Zoological Gardens, South Africa National Biodiversity Institution (SANBI), Pretoria, South Africa.

### 2.3 DNA extraction

Specimens were individually washed several times with distilled water to remove residual ethanol and patted dry with tissue to remove excess water. A 20 mg section of adult liver fluke tissue (*F. hepatica*) anterior to the ventral sucker but posterior to the pharyngeal area, thereby avoiding both the reproductive organs and the intestinal caeca, was used for DNA extraction. The DNA was extracted from the flukes using the ZR Genomic DNA™ Tissue MiniPrep (Zymo Research Corporation, California, United States) according to the manufacturer’s instructions. The DNA was quantified with a NanoDrop spectrophotometer and stored at −20 °C until needed.

### 2.4 Polymerase chain reaction and fragment analysis

The species of flukes were confirmed as *F. hepatica* based on a single-step multiplex PCR using Pepck primers Fh-Pepck-F: 5′-GATTGCACCGTTAGGTTAGC-3′; Fg-Pepck-F: 5′-AAAGTTTC TATCCCGAACGAAG-3′; and Fcmn-Pepck-R: 5′-CGAAAAT TATGGCATCAATGGG-3′ for *F. hepatica* and *F. gigantica*, respectively (38). Following the thermal cycling protocol as described by Shoriki et al. (38), *F. hepatica* and *F. gigantica* were identified by a band at 241 bp and 510 bp, respectively. The COI and ITS genes were amplified and sequenced using the primers FHCOI (forward: 5′-TTGGTTTTTTGGGCATCCT-3′) and RHCOI (reverse: 5′-AGG CCACCACCAAATAAAAGA-3′), and S30FE (forward: 5′-GTCGTAA CAAGGTTTCCGTA-3′) and S49E6 (reverse: 5′-TATGCTTAAA TTCAGCGGGT-3′) and protocol described by Haridwal et al. (25), respectively. Microsatellite markers (Fh_2, Fh_5, Fh_6, Fh_7, Fh_10, and Fh_12), developed for *F. hepatica* (39), were used to genotype individual flukes. These markers were selected based on their allelic size ranges to ensure coverage from the lowest to the highest observed allele sizes. Samples from the Limpopo province (*n* = 59) were excluded as they were predominantly *F. gigantica* and had suspected introgressed *Fasciola* forms. In addition, a sample containing DNA-free water in place of template DNA was included in all reactions as a negative control. Polymerase chain reactions (PCRs) were conducted using six sets of fluorescently labeled primers (Life Technologies, Carlsbad, United States), and the different alleles were discriminated based on allelic size range and fluorescence of individual primer pairs. Polymerase chain reaction amplification was conducted in a 10 μL reaction volume consisting of DreamTaq Green PCR Master Mix (Thermo Scientific, Massachusetts, United States), 1 μM of the fluorescent dye-M13 tagged forward primer and 0.5 μM of the reverse primer (Table 1), and 50 ng genomic DNA template. The cycling conditions described by Cwiklinski et al. (39) were followed with some modifications on annealing temperature (55 °C for Fh_2, Fh_5, Fh_7, Fh_10, and Fh_12). PCR products were analyzed by 2% agarose gel electrophoresis using SYBR® Safe DNA stain (Life Technologies, Carlsbad, United States). Bands were visualized under UV light. Amplicons were diluted 50-fold and 1 μL of this dilution multiplexed in 8.8 μL Hi-Di Formamide (Life Technologies, Carlsbad, United States) with 0.2 μL GeneScan LIZ500 size standards (Life Technologies, Carlsbad, United States) and run on an 3500xL Dx Genetic Analyzer (Applied Biosystems, Massachusetts, United States) and genotyped using GeneMapper® v. 4.0 (Applied Biosystems, Massachusetts, United States).

**Table 1:** Primer sequences and properties of *Fasciola hepatica* (Fh) microsatellite alleles.

Locus	Primer sequence (5′–3′)	Dye
Fh_2	F: 5′-TGTAAAACGACGGCCAGTTGAGAAACTGATTCACCGACTG-3′	VIC
	R: 5′-GAGCTTGTGCTCTCGGAACTA-3′	
Fh_5	F: 5′-TGTAAAACGACGGCCAGTCATCACCACTGTCTTCGATCA-3′	6-FAM
	R: 5′-CGAAGCATTGATAAGATTTCCA-3′	
Fh_6	F: 5′-TGTAAAACGACGGCCAGTACGTCCGTCCGTTAAGTGAG-3′	6-FAM
	R: 5′-TTTGAGGTCGACATCCTTCA-3′	
Fh_7	F: 5′-TGTAAAACGACGGCCAGTTGCACTCTAGCATGGTTTGG-3′	6-FAM
	R: 5′-AAGTCTTCAGTGCCCCTTCC-3′	
Fh_10	F: 5′-TGTAAAACGACGGCCAGTTTTAGTCGCGGAGCTACCAT-3′	NED
	R: 5′-CCACTTTCGTCATGCACATT-3′	
Fh_12	F: 5′-TGTAAAACGACGGCCAGTCCACGAGAAGTGGAATTCGT-3′	VIC
	R: 5′-GTAGGTCCACTCCCTGTCCA-3′	

The underlined terms highlight where the loci have similar nucleotides (Repeat motifs).

### 2.5 Data analysis

Statistical analyses for allele frequencies, F*is* and F*st* values, heterozygosity, and genetic distances were calculated on GenAlEx 6.51b2 (40) using the full dataset. Structure 2.3.4 (41) was used to detect population structure with default settings. Burn-in length was set at 100,000 and followed by 100,000 Markov Chain Monte Carlo repeats. The value of K was set at 1–10 and repeated 20 times. To determine the most appropriate value for K, Delta K was determined using the method proposed by Evanno et al. (42) and calculated using StructureSelector (43). To avoid bias due to sample size, the Gauteng population structure was analyzed separately as it had a higher sample size (84) compared to the other six provinces, which were combined (105) for analyses.

Genetic differentiation of *F. hepatica* populations across all six loci was assessed using the following parameters: total number of alleles (Na); (Ne); Information Index (I); He; Ho; unbiased expected heterozygosity (uHe), and F.

## 3 Results

### 3.1 Identification of fluke species

PCR amplification of the Pepck confirmed that all 189 adult *Fasciola* specimens were *F. hepatica*, as they produced diagnostic bands at 241 bp, consistent with *F. hepatica*. Sequence comparisons of the COI and ITS regions with GenBank references provided further confirmation of species identity.

### 3.2 Genotyping

All six microsatellite loci (Fh_2, Fh_5, Fh_6, Fh_7, Fh_10, and Fh_12) were successfully amplified across most of the 189\u00B0*F. hepatica* samples. A total of 146 samples (77.249%) yielded complete genotypes at all six loci. Amplification success per locus was as follows: Fh_2 (163/189), Fh_5 (160/189), Fh_6 (164/189), Fh_7 (164/189), Fh_10 (167/189), and Fh_12 (155/189) (Supplementary Table S1).

### 3.3 Genetic differentiation

A total of 277 alleles (Na) were detected from the seven *F. hepatica* populations, and the number of alleles per locus ranged from 2 (FH_7/FH_12) to 18 (FH_6), with an average of 7 alleles per locus. The number of effective alleles (Ne) ranged from 1.198 (FH_12) to 9 (FH_6), and the average was 3.568. Shannon’s Information index (I) for each locus ranged from 0.305 (FH_12) to 2.254 (FH_6), with an average of 1.390. This suggests that some loci have higher diversities than others. Observed heterozygosity (Ho) ranged from 0.182 to 1.000, with an overall mean of 0.653, while the values of expected heterozygosity (He) ranged from 0.165 to 0.899, with a grand mean of 0.665, indicating that there is variety in genetic diversity and population structure across the samples studied. The *F*-value ranged from −0.017 (FH_7) to 0.426 (FH_2) (Supplementary Table S2).

The within-population deficiency of heterozygosity as determined by F*is* ranged between −0.025 (FH_7) and 0.088 (FH_2) with an average of 0.015 for all loci. F*st* values varied from 0.064 (FH_5) to 0.107 (FH_2), with a mean of 0.076. The Nm value varied between 2.080 (FH_2) and 3.898 (FH_7) with an average of 3.173 in the whole population and across all loci (Table 2).

**Table 2:** F-statistics and estimates of migrants per generation (Nm) for each locus of populations of *Fasciola hepatica* from seven provinces of South Africa.

	Locus	F*is*	F*it*	F*st*	Nm
All populations	FH_2	0.088	0.185	0.107	2.080
	FH_5	0.013	0.077	0.064	3.641
	FH_6	0.085	0.162	0.084	2.728
	FH_7	−0.025	0.037	0.060	3.898
	FH_10	−0.032	0.035	0.065	3.588
	FH_12	−0.039	0.038	0.075	3.102
Mean		0.015	0.089	0.076	3.173
SEM		0.024	0.028	0.007	0.278

SEM, standard error of mean.

**Figure 1:**
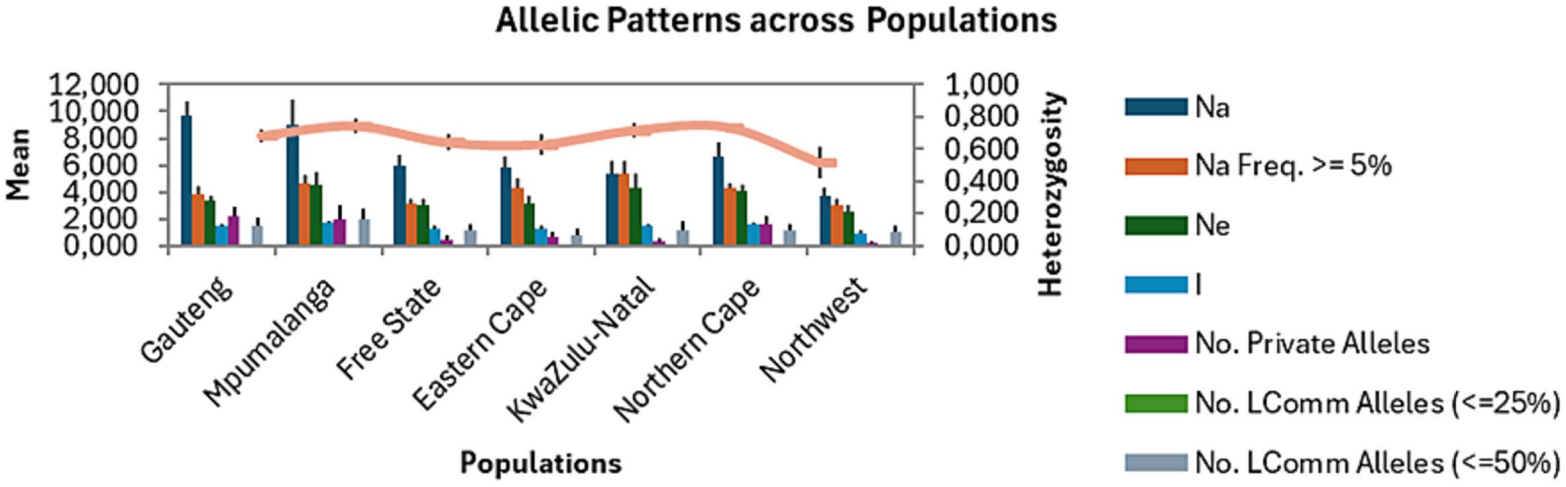
Allelic patterns of *Fasciola hepatica* populations from seven provinces of South Africa. Na, allele number; Na Freq. ≥ 5%, number of different alleles with a frequency ≥ 5%; Ne, effective number of alleles, I, Shannon’s Information index; LComm, Less Common.

The mean number of alleles varied from 3.667 to 9.667 (Figure 1). The highest number of alleles (*n* = 10) was found in the Gauteng population, which is not surprising as this province constituted of the highest number of samples. This was followed by Mpumalanga (*n* = 9), Northern Cape (*n* = 7), Free State (*n* = 6), Eastern Cape (*n* = 6), KwaZulu-Natal (*n* = 5), and North West (*n* = 4) (Table 3). Of the total number of alleles, 3% were private alleles (7.500), and the Gauteng, Mpumalanga, and Northern Cape populations had the highest number of private alleles (*n* = 2), followed by Eastern Cape (*n* = 1), Free State (*n* = 1), KwaZulu-Natal (*n* = 0.333), and North West (*n* = 0.167). The I value, which is an expression of population diversity in a particular habitat, was high in the Mpumalanga population (1.697) and low in the North West (0.951) population. The He in the populations ranged from 0.514 (North West) to 0.743 (Mpumalanga) (Tables 3, 4). The high values for I and He in Mpumalanga suggest greater genetic diversity, which may complicate control strategies due to enhanced adaptability or the potential emergence of drug-resistant genotypes.

**Table 3:** Genetic diversity indices of *Fasciola hepatica* among populations from seven provinces of South Africa.

Mean values			
Parameters measured	Gauteng	Mpumalanga	Free State	Eastern Cape	KwaZulu-Natal	Northern Cape	North West
Na	9.667	9.000	6.000	5.833	5.333	6.667	3.667
Na Freq. ≥ 5%	3.833	4.667	3.167	4.333	5.333	4.333	3.000
Ne	3.373	4.590	3.038	3.151	4.295	4.029	2.503
I	1.484	1.697	1.307	1.279	1.443	1.569	0.951
NPa	2.167	2.000	0.500	0.667	0.333	1.667	0.167
NCa (≤25%)	0.000	0.000	0.000	0.000	0.000	0.000	0.000
NCa (≤50%)	1.500	2.000	1.167	0.833	1.167	1.167	1.000
He	0.682	0.743	0.639	0.627	0.715	0.735	0.514
uHe	0.686	0.764	0.664	0.646	0.775	0.767	0.539
P %	100	100	100	100	100	100	100

Na, allele number; Na Freq. ≥ 5%, number of different alleles with a frequency ≥ 5%; Ne, effective number of alleles, I, Shannon’s Information index; NPa, number of private alleles; NCa, number of Less Comm Alleles; He,expected heterozygosity; uHe, unbiased expected heterozygosity; P %, percentage of polymorphic loci.

**Table 4:** Mean (±SE) of different genetic parameters in *Fasciola hepatica* populations from the seven sampled provinces of South Africa.

Population	Mean/SE	*N*	Na	Ne	I	Ho	He	uHe	F
Gauteng	Mean	84.000	9.667	3.373	1.484	0.621	0.682	0.686	0.088
	SE	0.000	1.116	0.394	0.114	0.036	0.039	0.039	0.016
Mpumalanga	Mean	18.000	9.000	4.590	1.697	0.812	0.743	0.764	−0.103
	SE	1.592	1.862	0.890	0.195	0.036	0.043	0.043	0.050
Free State	Mean	13.000	6.000	3.038	1.307	0.577	0.639	0.664	0.102
	SE	0.000	0.730	0.404	0.139	0.055	0.050	0.052	0.028
Eastern Cape	Mean	17.000	5.833	3.151	1.279	0.559	0.627	0.646	0.105
	SE	0.000	0.872	0.564	0.186	0.058	0.066	0.068	0.024
KwaZulu-Natal	Mean	6.500	5.333	4.295	1.443	1.000	0.715	0.775	−0.429
	SE	0.224	1.022	1.021	0.193	0.000	0.047	0.051	0.092
Northern Cape	Mean	12.167	6.667	4.029	1.569	0.561	0.735	0.767	0.247
	SE	0.167	1.054	0.472	0.114	0.071	0.029	0.030	0.072
North West	Mean	11.000	3.667	2.503	0.951	0.439	0.514	0.539	0.077
	SE	0.000	0.667	0.460	0.203	0.064	0.097	0.102	0.085

*N*, the number of samples; Na, no. of different alleles; Ne, no. of effective alleles; I, Shannon’s Information Index; Ho, observed heterozygosity; He, expected heterozygosity; uHe, unbiased expected heterozygosity; F, fixation index; SE, standard error of mean.

The Nei’s genetic distance as a pairwise population matrix was calculated based on allele frequencies. Genetic distances were generally lower between geographically proximate provinces (e.g., Free State and Eastern Cape: 0.061), while greater divergence was noted between more distant or isolated populations (e.g., Free State and North West: 0.360), reflecting potential barriers to gene flow (Table 5).

**Table 5:** Pairwise population matrix of Nei’s genetic distance among *Fasciola hepatica* from seven provinces of South Africa.

Population	Gauteng	Mpumalanga	Free State	Eastern Cape	KwaZulu-Natal	Northern Cape	North West
Gauteng	0.000						
Mpumalanga	0.106	0.000					
Free State	0.074	0.136	0,000				
Eastern Cape	0.075	0.079	0.061	0.000			
KwaZulu-Natal	0.242	0.259	0.318	0.286	0.000		
Northern Cape	0.186	0.186	0.191	0.182	0.289	0.000	
North West	0.302	0.226	0.360	0.260	0.225	0.179	0.000

### 3.4 Population structure

**Figure 2:**
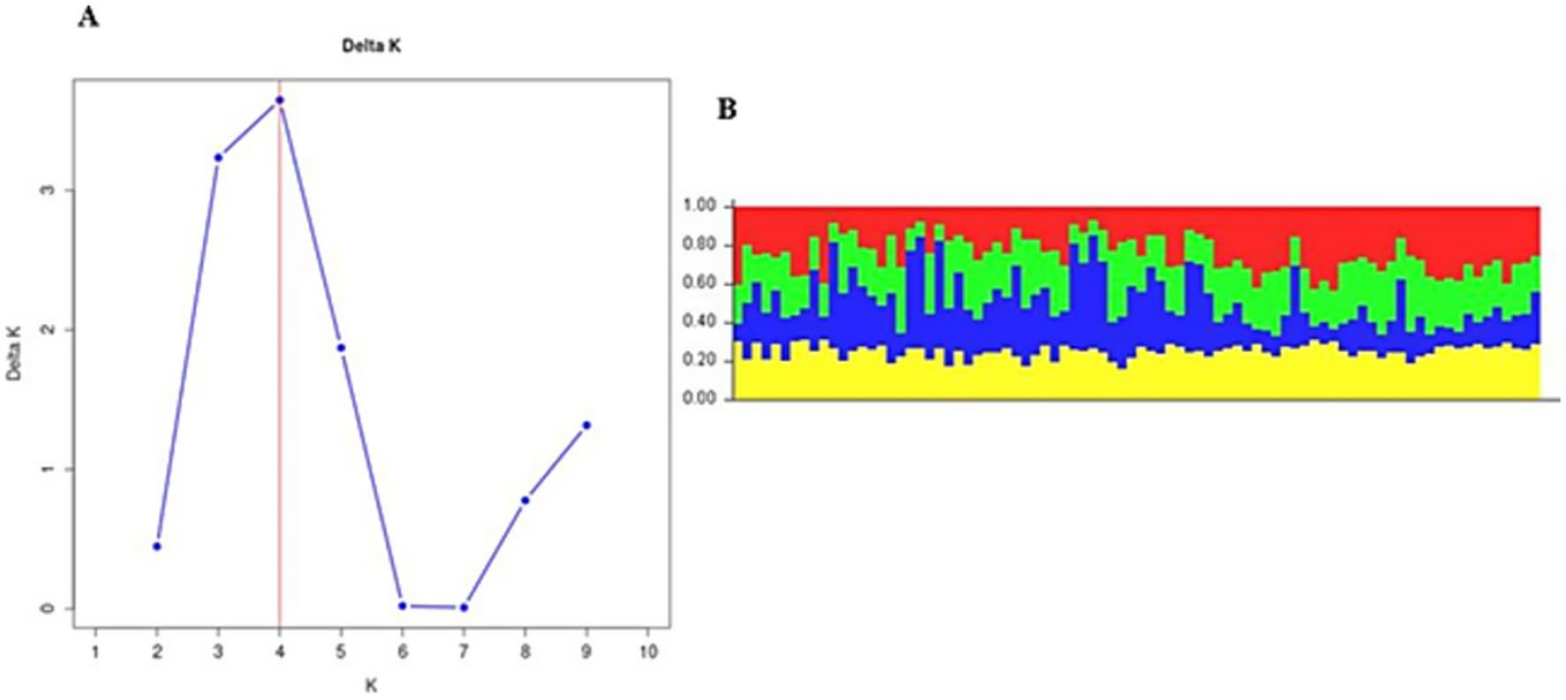
**(A)** Population structure analysis of *Fasciola hepatica* from the Gauteng province. **(B)** Bar plot from structure analysis.

**Figure 3:**
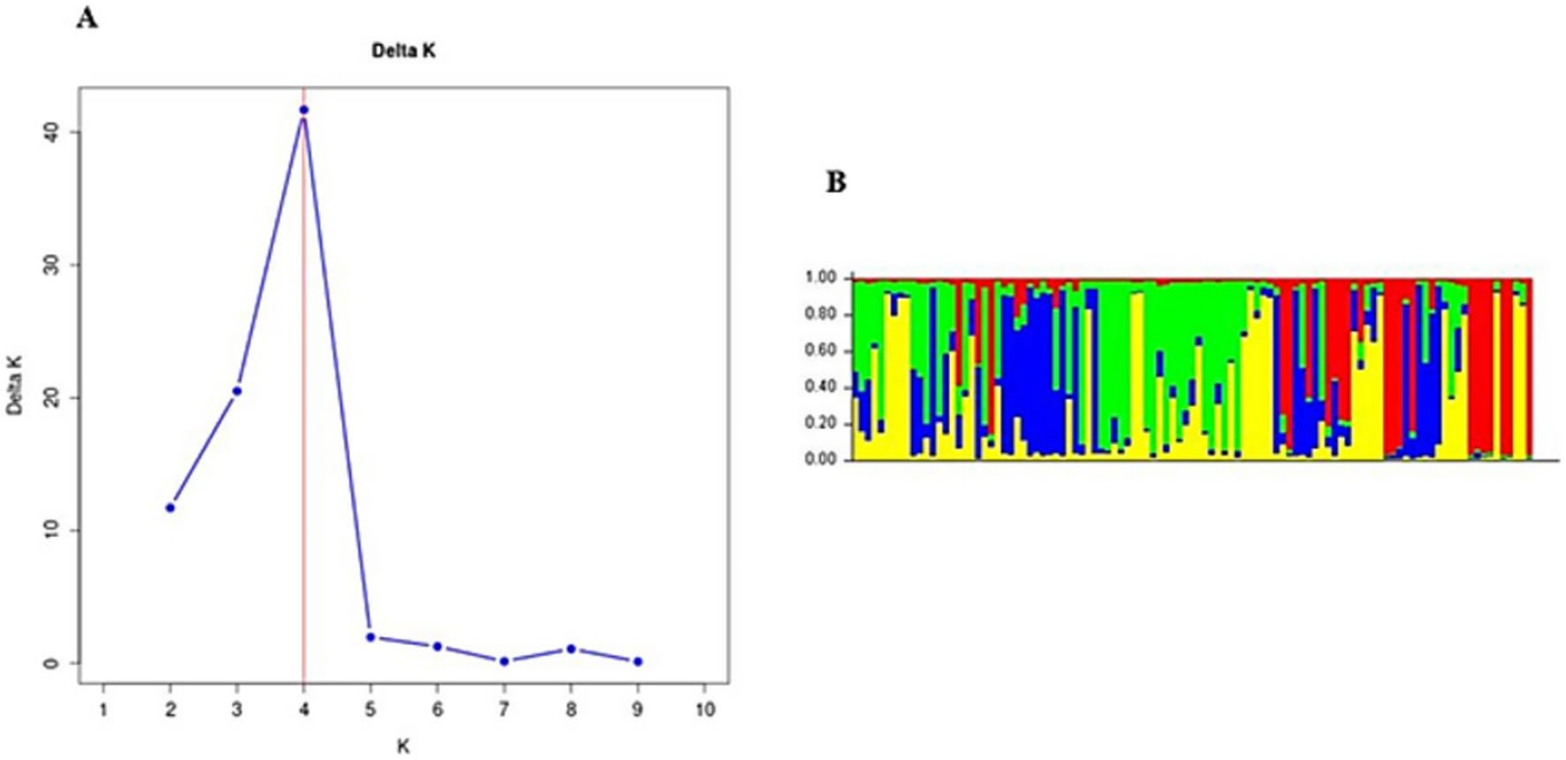
**(A)** Population structure analysis of *Fasciola hepatica* from the Mpumalanga, Free State, KwaZulu-Natal, Eastern Cape, Northern Cape, and North West provinces. **(B)** Bar plot from structure analysis.

**Figure 4:**
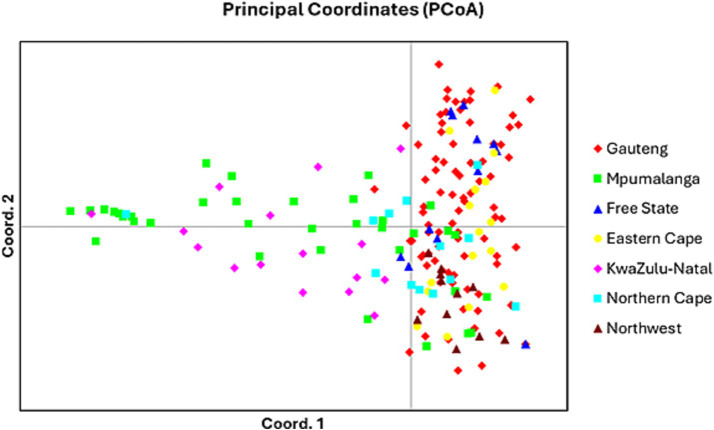
Principal coordinate analysis (PCoA) generated in GENALEX using Nei’s genetic distance matrix for all seven populations.

In Gauteng province, the results suggested that the most probable number of population clusters is K = 4. This is the value of K at which the estimated Ln probability peaks (−1857.245; SD = 127.063) and elevates (Figure 2A). For the other six provinces, the results also indicated K = 4 as the most likely number of population clusters (n = 4). At this value, the mean estimated Ln probability peaked (−1669.125; SD = 2.660) and elevated (Figure 3A). The iterations with the highest estimated Ln probability were chosen, and bar plots of the population clusters were produced (Figures 2B, 3B). Principal coordinate analysis also showed evidence of population clustering where *F. hepatica* populations in Mpumalanga, KwaZulu-Natal, and Northern Cape provinces spread across the plot, indicating high within-population diversity, while populations in Gauteng, Free State, Eastern Cape, and North West provinces showed a dense cluster on the right, suggesting stronger genetic relatedness among individuals in these provinces (Figure 4). Population structuring of *F. hepatica* in the Gauteng province and across the other six provinces of South Africa was supported by STRUCTURE analysis.

## 4 Discussion

Genetic diversity of a population can be reflected by various parameters, such as the effective number of alleles, Nei’s genetic distance, and I (44). High values of these parameters indicate the gene richness of the population (44). In this study, the average number of alleles from six pairs of polymorphic primers in seven *F. hepatica* populations was seven, which was higher than 1.330 and 4.900 that were reported by Robles-Pérez et al. (45) and Ohari et al. (46); however, it was lower than 15.330 as reported by Beesley et al. (47). The results further indicated variances between the Na at the microsatellite loci and the Ne. All populations showed values of Ne lower than Na, which implied uneven distribution of alleles within *F. hepatica* populations and that the populations have not reached the maximum variability, and this may be due to one of the alleles being more frequent than the other in the polymorphic loci (45).

At the population level, Mpumalanga, Gauteng, and Northern Cape populations showed the highest Na, Ne, and I, whereas these parameters were low in the Free State, Eastern Cape, KwaZulu-Natal, and North West provinces, which is typical of population bottlenecks occurring. Private alleles were recorded in the study populations, with Gauteng, Mpumalanga, and Northern Cape provinces recording the highest number of private alleles. The Free State, Eastern Cape, KwaZulu-Natal, and North West showed the lowest number of private alleles. The variation in the occurrence of private alleles may be as a result of differences in the agroecological climate of the provinces and the adaptation response of *F. hepatica* populations as alleles may be positively selected in one environment but remain neutral in others (48). This could be the case with the private alleles observed in Mpumalanga, which were higher than in the Northern Cape since the latter is mostly a desert. Previous studies conducted in desert climates have highlighted the low prevalence of fasciolosis (49) due to a lack of the preferred warm and wet conditions that are conducive for the survival and reproduction of the snail IHs (50).

While some private alleles may be functionally neutral, others could become relevant if linked to traits such as drug resistance or reproductive adaptation. This is essential in understanding the genetic diversity of *Fasciola* species since such alleles are recessive and might be associated with drug resistance genes, and the ability of *F. hepatica* to self-fertilize, which may contribute to an increased frequency of its occurrence (47). Therefore, the high numbers of private alleles observed in Mpumalanga and Gauteng suggest that these regions may serve as reservoirs of genetic diversity, potentially supporting long-term transmission of fasciolosis or harboring variants with altered susceptibility and hence affecting the effectiveness of control interventions. The North West province exhibited consistently low diversity data across all parameters. This province had one of the smallest sample sizes and is also underrepresented in studies on the occurrence of *Fasciola* (51). However, the presence of both snail IHs of *Fasciola* in this province (52) may also highlight the possible misrepresentation and underestimation of fasciolosis. Currently, there are no published reports from this province on the occurrence fasciolosis in cattle, specifically owned by smallholder farmers (51). The small sample sizes from some provinces, particularly the North West, may restrict the relevance of the diversity estimates. In addition, *F. gigantica* populations were not included due to unsuccessful microsatellite amplification, limiting comparative analysis and interpretation of the results.

Among the seven populations of *F. hepatica* included in our study, populations from Mpumalanga and KwaZulu-Natal provinces showed a negative *F*-value. This finding points to excess heterozygosity and low levels of inbreeding, as further evidenced by the Ho and uHe, both of which were higher than the He. In these two provinces, both *F. hepatica and F. gigantica* and the two snail IHs, *P. columella* and *Galba truncatula*, co-exist, and this scenario may play a role in the transmission and epidemiological dynamics of the *Fasciola* spp., resulting in selection pressure to favor genetic diversity over inbreeding (52).

A degree of inbreeding and population differentiation for *F. hepatica* in different provinces of South Africa was highlighted by the overall observed mean F*st* value of 0.076, indicating moderate genetic differentiation. This suggests a moderate level of genetic structure, which was also observed in populations from Europe, and this was attributed to the isolation of populations by distance and the effect of different ecological factors across regions (47). Furthermore, such a genetic structure can be influenced by varying climates, snail IH extinction and recolonization, and livestock management practices (53). Other studies have also observed moderate differentiation in parasite populations with relatively high host mobility, such as trematodes and cestodes, where host movement supported gene flow; however, there was some regional differentiation, which may be due to environmental or host-specific factors (54, 55). Similarly, the provinces studied are characterized by various climatic conditions and agroecological zones (56), which might explain the observed genetic structures. Nonetheless, the low mean F*is* value of 0.015 and slightly higher F*st* value of 0.076 indicated minimal inbreeding within and among populations.

Nm values >1 suggest adequate migration to prevent genetic drift (57), and the mean Nm value of 3.173 from this study suggests high gene flow across populations. This could possibly have reduced genetic differentiation, thus supporting the moderate F*st* observed. With *F. hepatica*, such gene flow may be facilitated by the movement of livestock or wildlife hosts between provinces (47), particularly in the commercial beef and dairy industry, where animals are sold and transported frequently. High gene flow can have substantial epidemiological implications, as it may aid the spread of advantageous alleles across populations, including those related to drug resistance or adaptation to local hosts (58). This has been reported in *F. hepatica* parasites, where gene flow among different regions supported the spread of drug resistance alleles (47).

Studies on *F. hepatica* in other parts of the world have reported variable levels of genetic structure, often influenced by ecological or anthropogenic factors such as host movement and environmental barriers. Studies in Europe and South America have reported that regions with intensive movement of livestock had lower F*st* values and high Nm (59), as observed in this study. The abattoirs where the samples were collected also confirmed a high movement of livestock from various provinces for slaughter (60), which may also occur between farms for breeding or fattening cattle. This strongly suggests that host-controlled gene flow is one of the factors that influence genetic structure in *F. hepatica*, regardless of geographic location, although local conditions may change this effect. The lowest genetic distances were observed between geographically closer provinces, such as Mpumalanga and Gauteng (0.106), and Eastern Cape and KwaZulu-Natal (0.286), suggesting that gene flow between these neighboring provinces may assist in maintaining genetic similarity (61), facilitated by swift trade or movement of livestock between them. Conversely, higher genetic distances were observed between North West and other provinces, such as Free State (0.360) and Gauteng (0.302), indicating a significant genetic differentiation. This indicates that geographic or ecological barriers might limit gene flow between these provinces (62), leading to more distinct genetic profiles for *F. hepatica* populations in these areas. These findings align with the expected influence of geographic distance and potential barriers on genetic structure in parasite populations, where proximity often facilitates gene flow. However, some moderately high genetic distances between geographically closer regions, such as KwaZulu-Natal and Northern Cape (0.289), could also suggest regional adaptations or limited livestock movement between these provinces. The observed moderate-to-high genetic differentiation has implications for control strategies as it indicates that genetic traits, including those related to resistance, may spread unevenly across the country.

The population structuring of *F. hepatica* across the Gauteng province and other provinces in South Africa, as revealed by STRUCTURE analysis, indicates distinct genetic clusters. STRUCTURE identified K = 4 as the optimal number of clusters both in Gauteng and across six other provinces, indicated by the peak in the log probability of data (LnP) values and an associated increase in standard deviation (42). For Gauteng specifically, a peak at K = 4 (LnP = −1857.245, SD = 127.063) suggests a complex genetic structure, whereas similar clustering patterns emerged for the combined provinces at K = 4 with LnP = −1669.125, SD = 2.660. Principal coordinate analysis further validates this structure, with Mpumalanga and KwaZulu-Natal showing broader distribution across the coordinate plot, indicating higher genetic diversity within populations. In contrast, the population from Gauteng province appears as a dense cluster, implying closer genetic relatedness among individuals (63, 64). This clustering may reflect ecological or historical factors affecting gene flow in the Gauteng province.

## 5 Conclusion

The population genetic analyses of *F. hepatica* from South Africa have demonstrated variability in allele frequency, heterozygosity, and genetic differentiation across provinces. The detection of different alleles per locus indicated a high level of polymorphism. This variability was further supported by the number of Ne that varied from the total number of alleles, and the mean I value that was greater than 1.000, highlighting significant allelic diversity across loci. The overall similarity between Ho and He values, along with low F*is* values, suggests a balanced genetic variation within populations with limited inbreeding. There was moderate genetic differentiation among populations, with a sufficient level of genetic exchange across populations. The variation in the Na and private alleles by province, particularly the high numbers in Gauteng and Mpumalanga, suggests that these areas may act as reservoirs of genetic diversity of *F. hepatica* infections in cattle in South Africa. Nei’s genetic distances showed that provinces such as Free State and Eastern Cape have closely related populations, whereas North West and Northern Cape populations exhibit higher levels of genetic divergence. The high genetic distances between *F. hepatica* populations of KwaZulu-Natal, Northern Cape, and North West and those of other provinces indicate distinct genetic structuring within these provinces. While this study provides important insights into the genetic structure of *Fasciola hepatica* populations in South Africa, several limitations should be acknowledged. The uneven sample distribution across provinces, with overrepresentation from Gauteng, may have influenced estimates of genetic diversity and differentiation. Samples from Limpopo were excluded due to the predominance of *F. gigantica* and suspected hybrid forms, thereby limiting the geographic and species-level scope of the analysis. The use of only six microsatellite markers, although informative, may have limited the resolution of population structure. Future studies should aim for more balanced and longitudinal sampling across provinces and expand the panel of genetic markers to better understand the evolutionary and epidemiological dynamics of *Fasciola* spp. in South Africa.

## Data Availability

The original contributions presented in the study are included in the article/Supplementary material, further inquiries can be directed to the corresponding author.
