# Supplementary material for: Pilot study on the population genetics structure of Fasciola hepatica from seven provinces of South Africa
Source: Front Vet Sci. 2025 Sep 11;12:1659523. doi: 10.3389/fvets.2025.1659523 (PMC12461863; doi:10.3389/fvets.2025.1659523)
Supplement: Supplementary file 1 [file Table_1.docx]

Supplementary Material

# Supplementary Table

Supplementary Table S1. Summary statistics for the microsatellite panel based on 189 *Fasciola hepatica* from cattle in South Africa.

| Pop | Locus | N | Na | Ne | I | Ho | He | uHe | F |
| --- | --- | --- | --- | --- | --- | --- | --- | --- | --- |
| Gauteng | FH_2 | 84 | 12,000 | 3,375 | 1,590 | 0,643 | 0,704 | 0,708 | 0,086 |
|  | FH_5 | 84 | 8,000 | 2,221 | 1,261 | 0,500 | 0,550 | 0,553 | 0,090 |
|  | FH_6 | 84 | 14,000 | 4,038 | 1,765 | 0,631 | 0,752 | 0,757 | 0,161 |
|  | FH_7 | 84 | 8,000 | 3,408 | 1,456 | 0,655 | 0,707 | 0,711 | 0,073 |
|  | FH_10 | 84 | 9,000 | 4,780 | 1,762 | 0,750 | 0,791 | 0,796 | 0,052 |
|  | FH_12 | 84 | 7,000 | 2,416 | 1,067 | 0,548 | 0,586 | 0,590 | 0,066 |
| Mpumalanga | FH_2 | 19 | 8,000 | 3,297 | 1,473 | 0,842 | 0,697 | 0,716 | -0,209 |
|  | FH_5 | 16 | 7,000 | 4,163 | 1,655 | 0,750 | 0,760 | 0,784 | 0,013 |
|  | FH_6 | 21 | 18,000 | 8,167 | 2,508 | 0,905 | 0,878 | 0,899 | -0,031 |
|  | FH_7 | 17 | 6,000 | 3,247 | 1,386 | 0,882 | 0,692 | 0,713 | -0,275 |
|  | FH_10 | 23 | 9,000 | 6,224 | 1,970 | 0,826 | 0,839 | 0,858 | 0,016 |
|  | FH_12 | 12 | 6,000 | 2,441 | 1,191 | 0,667 | 0,590 | 0,616 | -0,129 |
| Free State | FH_2 | 13 | 5,000 | 2,113 | 1,043 | 0,462 | 0,527 | 0,548 | 0,124 |
|  | FH_5 | 13 | 4,000 | 1,867 | 0,844 | 0,385 | 0,464 | 0,483 | 0,172 |
|  | FH_6 | 13 | 9,000 | 4,225 | 1,731 | 0,692 | 0,763 | 0,794 | 0,093 |
|  | FH_7 | 13 | 6,000 | 3,130 | 1,400 | 0,692 | 0,680 | 0,708 | -0,017 |
|  | FH_10 | 13 | 7,000 | 4,122 | 1,619 | 0,692 | 0,757 | 0,788 | 0,086 |
|  | FH_12 | 13 | 5,000 | 2,770 | 1,209 | 0,538 | 0,639 | 0,665 | 0,157 |
| Eastern Cape | FH_2 | 17 | 5,000 | 2,369 | 1,064 | 0,529 | 0,578 | 0,595 | 0,084 |
|  | FH_5 | 17 | 4,000 | 2,181 | 0,983 | 0,471 | 0,542 | 0,558 | 0,131 |
|  | FH_6 | 17 | 8,000 | 3,482 | 1,542 | 0,647 | 0,713 | 0,734 | 0,092 |
|  | FH_7 | 17 | 7,000 | 3,932 | 1,609 | 0,588 | 0,746 | 0,768 | 0,211 |
|  | FH_10 | 17 | 8,000 | 5,352 | 1,840 | 0,765 | 0,813 | 0,838 | 0,060 |
|  | FH_12 | 17 | 3,000 | 1,592 | 0,634 | 0,353 | 0,372 | 0,383 | 0,051 |
| KwaZulu-Natal | FH_2 | 7 | 6,000 | 5,158 | 1,710 | 1,000 | 0,806 | 0,868 | -0,241 |
|  | FH_5 | 7 | 4,000 | 2,800 | 1,171 | 1,000 | 0,643 | 0,692 | -0,556 |
|  | FH_6 | 6 | 10,000 | 9,000 | 2,254 | 1,000 | 0,889 | 0,970 | -0,125 |
|  | FH_7 | 6 | 4,000 | 3,429 | 1,309 | 1,000 | 0,708 | 0,773 | -0,412 |
|  | FH_10 | 7 | 5,000 | 3,063 | 1,296 | 1,000 | 0,673 | 0,725 | -0,485 |
|  | FH_12 | 6 | 3,000 | 2,323 | 0,918 | 1,000 | 0,569 | 0,621 | -0,756 |
| Northern Cape | FH_2 | 12 | 4,000 | 3,646 | 1,340 | 0,417 | 0,726 | 0,757 | 0,426 |
|  | FH_5 | 12 | 8,000 | 3,032 | 1,536 | 0,417 | 0,670 | 0,699 | 0,378 |
|  | FH_6 | 12 | 11,000 | 5,878 | 2,044 | 0,667 | 0,830 | 0,866 | 0,197 |
|  | FH_7 | 13 | 5,000 | 4,024 | 1,500 | 0,615 | 0,751 | 0,782 | 0,181 |
|  | FH_10 | 12 | 7,000 | 4,800 | 1,712 | 0,833 | 0,792 | 0,826 | -0,053 |
|  | FH_12 | 12 | 5,000 | 2,796 | 1,280 | 0,417 | 0,642 | 0,670 | 0,351 |
| Northwest | FH_2 | 11 | 5,000 | 3,667 | 1,425 | 0,455 | 0,727 | 0,762 | 0,375 |
|  | FH_5 | 11 | 3,000 | 1,449 | 0,576 | 0,364 | 0,310 | 0,325 | -0,173 |
|  | FH_6 | 11 | 6,000 | 3,781 | 1,511 | 0,545 | 0,736 | 0,771 | 0,258 |
|  | FH_7 | 11 | 2,000 | 1,936 | 0,677 | 0,455 | 0,483 | 0,506 | 0,060 |
|  | FH_10 | 11 | 4,000 | 2,988 | 1,212 | 0,636 | 0,665 | 0,697 | 0,043 |
|  | FH_12 | 11 | 2,000 | 1,198 | 0,305 | 0,182 | 0,165 | 0,173 | -0,100 |
